# Real-Time Monitoring Method and Circuit Based on Built-In Reliability Prediction

**DOI:** 10.3390/mi16010004

**Published:** 2024-12-24

**Authors:** Wenke Ren, Yanning Chen, Xiaoming Li, Xinjie Zhou, Baichen Song, Tianci Chang

**Affiliations:** 1 School of Microelectronics, Xidian University, Xi’an 710071, China; 2 Beijing Smartchip Microelectronics Technology Company Limited, Beijing 100089, China

**Keywords:** real-time monitoring, reliability prediction, on-chip monitor, parameter degradation

## Abstract

The failure of different chips under working conditions is influenced by various stress states such as different voltages, temperatures, stress durations, and stress variations. Therefore, the failure time has a great degree of dispersion, and similar chips may exhibit different failure mechanisms due to variations in their working environments. This paper proposes three system-on-chip reliability failure prediction unit circuits: the time-dependent dielectric breakdown prediction circuit, the negative bias temperature instability prediction circuit, and the hot carrier injection prediction circuit. These circuits are embedded within the main chip, enabling real-time failure prediction and reliability mechanism diagnosis in the same working environment as the main chip. The three reliability failure prediction circuits are compact and energy efficient, allowing for their integration into a system on a chip as IP cores that provide early warning signals before system-on-chip failure. Compared to traditional reliability prediction methods, this approach offers the advantages of accurately identifying failure mechanisms, predicting failure times, and enabling real-time online monitoring.

## 1. Introduction

As the size of transistors continues to shrink and integrated circuit manufacturing processes advance, the risk of circuit components experiencing various inevitable degradation and failure effects also increases [1,2,3]. NBTI (negative bias temperature instability), TDDB (time-dependent dielectric breakdown), and HCI (hot carrier injection) are the primary factors causing transistor degradation [4,5]. These effects will change the electronic structure of a transistor, resulting in issues such as critical voltage changes and leakage current increases, thereby affecting the stability and reliability of the entire integrated circuit [6,7]. SoC (system-on-chip) reliability issues are typically characterized by multiple failure mechanisms, complicating reliability verification. Thus, predicting the lifespan of circuit systems and identifying faults has become a focal point in current circuit reliability analysis.

**Figure 1:**
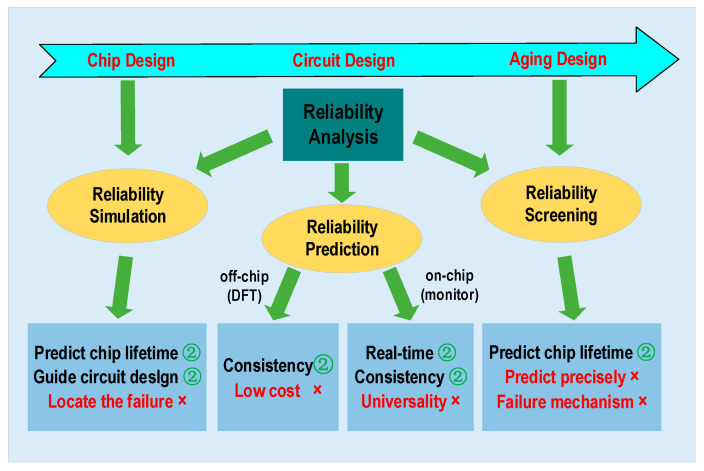
Reliability analysis methods for different design stages.

The current reliability research can be divided into three primary aspects based on event execution time: reliability simulation, reliability prediction, and reliability screening. Figure 1 illustrates that reliability prediction can be divided into two categories: off-chip reliability monitoring and on-chip reliability monitoring. Reliability simulation involves modeling the lifespan of the chip by assessing the reliability of devices and circuits during the early chip design stage. References [8,9,10] employ reliability simulation tools to model failure mechanisms at the circuit level, thereby simulating the drift of critical parameters such as threshold voltage and mobility. These parameters are crucial for predicting the lifespan of circuits. However, the accuracy of these simulations is heavily reliant on the artificially established accelerated aging conditions, which makes it challenging to precisely forecast the exact failure time of a chip. Reliability prediction is performed during circuit design through the inclusion of a DFT (Design for Testability) in the circuit for off-chip testing. Alternatively, a life prediction unit can be incorporated as IP (Intellectual Property) into the circuit, which undergoes the same operating stresses as the main circuit. This causes the life prediction unit to age faster and trigger an alarm on the chip. The off-chip testing method, as described in reference [11], involves aging tests on the target circuit using an FPGA (Field-Programmable Gate Array). However, this method incurs significant testing costs and is impractical for large-scale application in industrial-grade chips. Reference [12] proposed an on-chip reliability sensor that quantifies the frequency variations of digital circuits using a VCO (Voltage-Controlled Oscillator) to detect the extent of circuit degradation. This method enables real-time on-chip monitoring, but it cannot identify the specific mechanisms that cause the circuit degradation. References [13,14] suggest an on-chip aging monitor for NBTI based on SRAM (Static Random-Access Memory), respectively. However, it lacks generalizability and may result in significant mistakes when utilized in SoCs due to PVT (process, voltage, and temperature) deviation and variations in operating environments. References [15,16] proposed two circuit architectures for monitoring critical path delays. However, the approach in reference [15] was highly targeted, being specifically designed to monitor the critical path of SRAM in advanced process nodes. Reference [16] focused on detecting reductions in slack time along critical paths, using these reductions as an alarming signal for system degradation. Reliability screening can be conducted during the test design stage or aging design stage. Reference [17] introduces a reliability screening approach based on rapid on-chip zero-aging prediction, which facilitates the forecasting of aging effects for HCI and NBTI by monitoring the delay of critical paths. References [18,19] employ machine learning techniques to assess the reliability of circuits. By extracting specific parameters from the circuit and using trained samples, these studies aim to ascertain the remaining useful lifetime of the chip. Despite the availability of a substantial number of training samples, the discreteness in working environments and stress conditions for each chip complicates the simulation of the chip’s operating conditions. This leads to a significant degree of dispersion in the predicted remaining lifespan of the chips.

In previous studies, whether predicting lifetime through reliability simulation before chip design or accelerated aging after design completion, it has not been possible to accurately target each individual chip. Although some predictive circuits provide pre-emptive forecasting, they are typically designed for specific modules, such as SRAM or digital circuits. Therefore, designing a monitoring circuit IP that can be applied to failure prediction in both analog and digital circuits represents a meaningful endeavor.

This paper respectively proposes an on-chip reliability prediction unit circuit or monitoring logic for failures caused by TDDB, NBTI, and HCI. Since the monitoring circuit is embedded within the system circuit, it ensures a high degree of consistency with the main circuit throughout the processes of production, manufacturing, storage, and use. If the working environments and stress parameters of these circuits are identical, their failure rates will also align closely. This approach overcomes the limitations of traditional reliability simulations and lifespan predictions, which can only be conducted before or after the event, achieving the goal of real-time on-chip prediction of circuit lifespan.

## 2. Degradation Mechanism and Monitoring Principle

**Figure 2:**
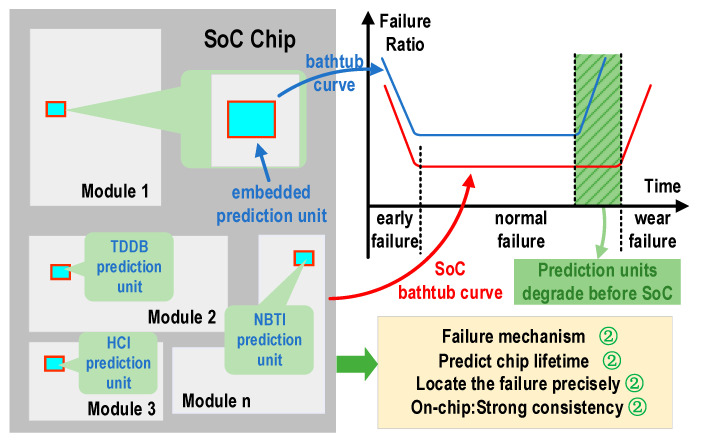
Embedded on-chip real-time monitoring method.

This paper presents a novel reliability prediction and assessment technique for system failures caused by three types of reliability issues in circuits. As shown in Figure 2, the three prediction units are embedded in various modules of the SoC, including the TDDB prediction unit, the NBTI prediction unit, and the HCI prediction unit. Due to their consistent working environment with the SoC, these units can accurately predict the failure time and precisely identify the failure mode. The bathtub curve indicates that in the process of reliability evaluation and failure prediction, the prediction circuit fails prior to the main circuit. As the main circuit approaches failure, the prediction circuit generates an alarm signal and indicates the mechanism of failure.

### 2.1. TDDB Degradation Prediction Principle

Based on the time of breakdown, gate oxide breakdown can be categorized into two types: intrinsic breakdown and time dependent dielectric breakdown (TDDB). Intrinsic breakdown occurs when the electric field strength applied across the gate oxide layer reaches or exceeds the critical field strength that the gate oxide can withstand over a short period, forming a conductive path and resulting in breakdown. Conversely, when the external electric field is below the intrinsic breakdown field strength of the gate oxide layer, the application of electrical stress can still lead to the accumulation of defects within the gate oxide layer. Over time, this defect accumulation can result in the formation of a conductive path, leading to TDDB [20,21]. Due to the extended duration of TDDB under normal operating conditions, it is necessary to apply appropriate voltage–temperature stress to induce failure in the prediction unit within a short period. This accelerates the lifetime testing process, allowing for the use of extrapolation methods to determine the lifespan of the chip under normal operating conditions. The definition of the ALF (Accelerated Life Factor) is as follows:

(1) $$ALF=\frac{T_{normal}}{T_{stress}}$$

where $T_{stress}$ represents the lifespan measured under stress conditions, and $T_{normal}$ represents the extrapolated lifespan under normal operating conditions. The factors influencing the lifespan include the gate oxide area, electrical stress, temperature stress, etc. Since the acceleration factors resulting from each influencing factor are independent, the total acceleration factor can be expressed as the product of these individual acceleration factors. Using the Arrhenius model to describe the relationship of the electrical field, the acceleration factor is expressed as follows:

(2) $$ALF\left(V\right)=exp\left(z\cdot \left|V_{stress}-V_{use}\right|\right)$$

where z denotes the voltage acceleration constant, typically ranging from 0.5 to 1.0, $V_{stress}$ signifies the stress voltage applied during the aging process, and $V_{use}$ is the gate voltage under normal operating conditions. Additionally, the gate oxide breakdown monitoring circuit needs to be embedded within the circuit under testing, sharing the same gate oxide dielectric thickness and temperature stress as the main circuit. Therefore, it is only necessary to consider the gate oxide dielectric area and electrical stress during the design process.

The TDDB prediction circuit proposed in this paper utilizes MOS capacitors for monitoring and includes modules such as the detection voltage source, stress voltage source, current-limiting resistor, MOS capacitor array, and a latch. In the TDDB prediction circuit, stress voltage is applied to the MOS capacitor array, causing the MOS capacitors to fail earlier than they would under normal use conditions. When one of the capacitors in the MOS capacitor array experiences gate oxide breakdown, the voltage across the current-limiting resistor will suddenly drop. The latch detects this voltage decrease and subsequently pulls the output low. Voltage stress is applied through the circuit structure, and the area of the dielectric gate oxide is adjusted by varying the number of MOS capacitors in the array. As the gate oxide breakdown monitoring circuits are embedded into the circuits under testing, they have the same gate oxide thickness and operate in the same environment. The monitoring circuits and the circuits under testing are exposed to the same PVT conditions, indicating a significant level of consistency.

### 2.2. NBTI Degradation Prediction Principle

In the reliability of integrated circuits, NBTI is frequently a crucial factor to be considered. This is because the NBTI effect is very prominent in PMOS transistors and exerts a substantial influence on circuit performance. The typical conditions for NBTI effects are as follows: within a temperature range of 373 K to 523 K and with a gate oxide electric field strength $E_{OX}<6\;MV/cm$. When the PMOS transistor is under stress, the gate-to-source voltage is negatively biased, causing the formation of interface traps at the silicon-oxide layer (Si-SiO_2_) interface. This leads to changes in the threshold voltage, a decrease in transistor transconductance performance, a reduction in the drain current, and a decrease in the driving capability [22,23,24]. The relationship between the threshold voltage shift caused by NBTI effects and time is as follows:

(3) $$\Delta V_{T}=A\exp(\beta V_{G})\exp(-E_{a}/kT)t^{n}$$

where $V_{G}$ represents the gate voltage, A and β are constant, k is the Boltzmann constant, T is the temperature, and $E_{a}$ is the activation energy. The drift of the threshold voltage changes exponentially over time, with the value of n ranging between 0.2 and 0.3. From Formula (3), it can be observed that the absolute value of the threshold voltage increases with increasing time. Therefore, this paper employs a method that directly measures the shift in threshold voltage to predict whether the main circuit has experienced NBTI degradation.

The method for measuring the threshold voltage of a PMOS transistor proposed in this paper requires that the gate and drain be short circuited, causing the transistor to operate in the saturation region, and a constant current is applied. Under these conditions, the drain-source voltage is as follows:

(4) $${V_{DS}=V}_{GS}=V_{th}+\sqrt{\frac{2I_{DS}\left(1+\delta \right)}{\mu C_{ox}\frac{W}{L}}}$$

(5) $$\delta =\frac{\gamma }{2\sqrt{\left(2\Psi_{F}-V_{BS}\right)}}$$

where $V_{th}$ is the initial threshold voltage, $C_{ox}$ is the gate oxide capacitance per unit area, μ is the carrier mobility, $I_{DS}$ is the constant saturation drain current applied, WL is the PMOS width-to-length ratio, γ is the body effect coefficient, $\Psi_{F}$ is the Fermi potential of the substrate silicon, and $V_{BS}$ is the bulk-source voltage. According to Formulas (4) and (5), when the threshold voltage experiences a shift due to NBTI stress, the absolute value of this shift is equal to the change in voltage at the PMOS drain terminal (${∆V}_{DS}=V_{th}$ because $I_{DS}$ is constant). The NBTI monitoring circuit proposed in this paper measures the $V_{th}$ variation by measuring the $V_{DS}\;$ of two gate–drain short-circuit PMOSs. We apply higher voltage stress to the PMOS within the test unit, causing it to reach the critical failure point earlier than the main circuit. The circuit implementation proposed in this paper involves monitoring two transistors of identical dimensions, ensuring that the only variable is the stress voltage. When process variations occur, the variations affecting both transistors due to process changes are consistent.

### 2.3. HCI Degradation Prediction Principle

In CMOS technology, when the drain-source voltage of the transistor is high, some of the carriers in the channel can gain sufficient energy to collide and ionize near the drain junction. These high-energy carriers and the carriers generated by impact ionization are referred to as hot carriers. The impact of hot carriers on NMOS is characterized by a decrease in drive performance, as well as changes in the threshold voltage, transconductance in the linear region, and the driving current in the saturation region. For digital logic gates, the HCI effect not only reduces device performance but also impacts on the circuit’s delay [25,26,27]. Taking an inverter as an example, if we disregard the degradation of the drain current in the saturation region, then the relationship between the degradation ratio of the delay in the linear region and the degradation ratio of the drain current can be expressed as follows:

(6) $$\frac{\Delta \tau }{\tau }=K\cdot \frac{\left(\frac{\Delta I_{DS}}{I_{DS}}\right)}{1-\left(\frac{\Delta I_{DS}}{I_{DS}}\right)}$$

where K is a constant, with a typical value ranging between 0.6 and 0.7, Δττ represents the degradation ratio of the delay in the linear region, and ΔIDSIDS denotes the degradation ratio of the drain current in the linear region. According to Formula (6), there is a linear relationship between the output delay of a CMOS inverter and the degradation of the drain current. Consequently, the delay of the inverter circuit can be used to reflect the drain current degradation of logic gates. Simultaneously, the output frequency of a ring oscillator composed of multiple stages of inverters is inversely proportional to the delay of the inverters. Therefore, this paper employs two ring oscillators, each constructed from inverters of different types, as the fundamental unit to reflect the circuit’s propagation delay. Additionally, other circuit modules are designed to detect variations in the frequency. The frequency of the ring oscillator (f) is inversely proportional to the delay (τ) of the inverter. The following formula reflects the relationship between the degradation ratio of the ring oscillator’s frequency and the degradation ratio of the drain current:

(7) $$\frac{\Delta f}{f}=K\cdot \frac{\left(\frac{\Delta I_{DS}}{I_{DS}}\right)}{\left(\frac{\Delta I_{DS}}{I_{DS}}\right)-1}$$

where Δff represents the frequency degradation ratio of the ring oscillator. The dual ring oscillation monitoring circuit designed in this paper is based on the above principle and generates the reference frequency by protecting the transistor of the inverter, which is seriously affected by HCI effect, thus avoiding the reference frequency from being changed by external signals. At the same time, when counting the frequency, this paper designs a multi-fold frequency division of the reference frequency to increase the counting value to improve the frequency detection accuracy. The designed prediction circuit detects the frequency degradation using digital logic. When the degradation reaches a failure threshold, the circuit outputs the alarm signal “ALM”.

## 3. Implementation of Built-In Reliability Monitoring Circuits

### 3.1. TDDB Monitoring Circuits

**Figure 3:**
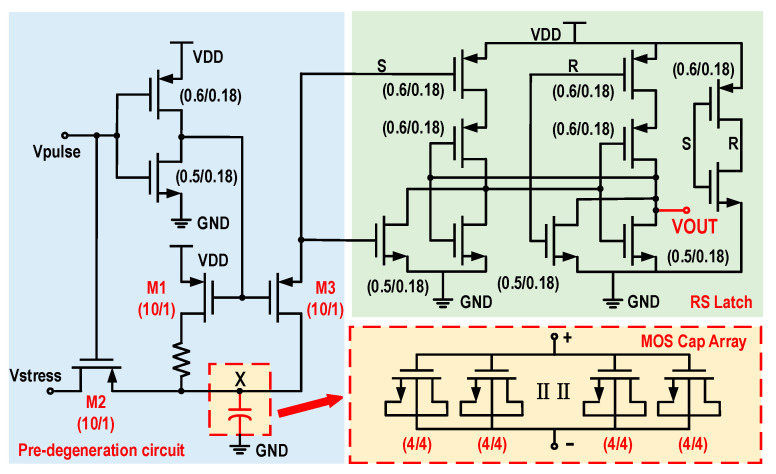
TDDB prediction circuit structure diagram; unit: μm.

The TDDB prediction circuit structure, as shown in Figure 3, applies the gate oxide stress voltage $V_{stress}$ to the MOS capacitors through the MOS switch transistor M2, with these capacitors connected in parallel. Among them, M1, M2, and M3 switching transistors, in order to achieve low on-resistance, have a size of W/L = 10 μm/1 μm; since the transistors in the MOS capacitor array need to be consistent with the size of the transistors in the SoC to be tested, in this paper, a size of W/L = 4 μm/4 μm is used to accelerate the breakdown of the gate oxide layer; the other transistors in the circuit are used to deal with the digital logic, and therefore, we use standard transistors. The stress voltage $V_{stress}$ is slightly higher than the supply voltage, indicating that the gate oxide dielectric of the MOS capacitor array is under greater stress. Consequently, when embedded into the main circuit, the MOS capacitor array will degrade more rapidly than the circuit being monitored. The read voltage is a periodic pulse signal. During its high level, it controls the conduction of switch transistors M1 and M3. Currently, the voltage value at node X is read, and the latch determines whether the MOS capacitor array has experienced dielectric rupture. During the low level, it controls the conduction of switch transistor M2, which corresponds to the stress application state. If the MOS capacitor array experiences a time-dependent dielectric breakdown, there will be a significant change in the voltage at node X. The latch monitors and latches the voltage at node X. If the voltage at node X is high, it implies that both the prediction circuit and the circuit being monitored are functioning normally, and the latch outputs a logic of “1” (VOUT = 1). Conversely, if there is a dielectric breakdown of the gate oxide in any of the MOS capacitors, the voltage at node X drops to low, indicating that the prediction circuit has failed, and the latch outputs a logic of “0” (VOUT = 0).

### 3.2. NBTI Monitoring Circuits

**Figure 4:**
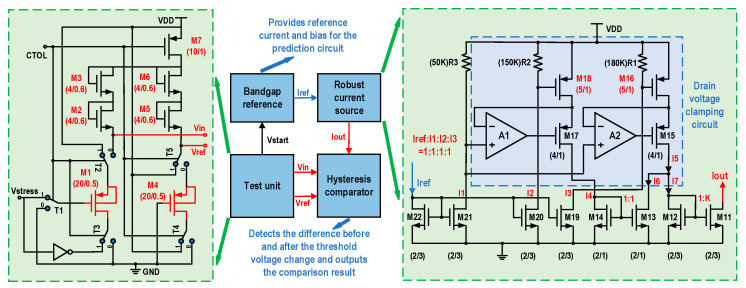
NBTI prediction circuit structure diagram; unit: μm.

As shown in Figure 4, the NBTI prediction circuit mainly consists of the following four components: a test unit, a bandgap reference source, a PVT compensation circuit, and a hysteresis comparator. Each component plays a critical role in the overall functionality of the circuit, ensuring the accurate prediction of NBTI effects under various operating conditions.

In the test unit, two identical PMOS transistors (M1 and M4) are selected and placed in two identical branches. One of them (M1 in the figure) is subjected to stress from the NBTI effect to measure the change in the threshold voltage. The other (M4 in the figure) has a circuit structure consistent with the previous branch but does not apply voltage stress, serving to measure and maintain the initial threshold voltage of the PMOS. This comparative approach allows for the direct observation of the impact of NBTI stress on the threshold voltage of the PMOS transistors (M1 and M4 need to be consistent with the size of the transistors in the SoC to be tested; in this paper, a size of W/L = 20 μm/0.5 μm is used to accelerate the degradation during testing).M2, M3, M5, and M6 are responsible for providing saturation drain currents for M1 and M4, and it is only necessary that the four transistors are of the same size and reasonable values, which, in this paper, are all 4 μm/0.6 μm. CTOL is a pulse signal that controls the switching between the stress application state and detection state. When CTOL is low, all switches are connected to the “0” terminal. The gate and drain of M1 are short circuited to ground, and a constant current is applied to its source, causing it to be in a saturated state. At this point, the source voltage of M1 is detected. When CTOL is high, all switches are connected to the “1” terminal. In this state, the gate and source of M1 are subjected to the stress of $V_{stress}$, causing the absolute value of its threshold voltage to continue increasing. The increase in the threshold voltage of M1 can be reflected in the circuit through the voltage at the source terminal. The change in the source-to-drain voltage of M1 is equivalent to the change in the threshold voltage. During the transition of the CTOL signal, the M4 in the other branch is not subjected to stress degradation, allowing its source-to-drain voltage to serve as a reference for the change in the threshold voltage of M1. The hysteresis comparator is utilized to compare the difference between the drain-source voltage of M1(Vin) and the reference voltage (Vref). If Vin exceeds Vref by a certain threshold, the hysteresis comparator outputs a low level and locks (the flag signal becomes low), indicating that the M1 transistor has experienced NBTI degradation.

The bandgap reference source and the hysteresis comparator are designed with reference to the structure presented in [28]. The relationship between the hysteresis voltage and the bias current is as follows:

(8) $$V_{HYS}\propto \sqrt{\frac{I_{ref}}{\mu C_{OX}}}$$

where $I_{ref}$ is the bias current of the hysteresis comparator, μ is the carrier mobility, and $C_{OX}$ is the gate oxide capacitance. These parameters are related to the manufacturing process or temperature [28]. Since the threshold voltage shift is minor and influenced by process variations, the power supply voltage, and temperature, compensation for the bias current is necessary to ensure accurate monitoring and prediction of the NBTI effect. In this paper, a PVT-compensated hysteresis comparator has been designed based on the circuit structure. This comparator generates a robust current bias to compensate for the effects of PVT variations on its operation. As shown in Figure 4, the current difference between two MOS transistors (M16 and M18, both 5 μm/1 μm in size) operating in the linear region is used to generate a compensation current related to the mobility and gate capacitance. This compensation current is then applied to the tail current end of the hysteresis comparator to compensate for the process- and temperature-related components in the hysteresis voltage, thereby creating a high-precision hysteresis comparator. M16 and M18 operate in the saturation region, and their current equations are as follows:

(9) $$\left\{\begin{array}{c} I_{\mathrm{DM}16}=\mu C_{ox}\frac{W_{16}}{L_{16}}((V_{\mathrm{GSM}16}-V_{\mathrm{TH}})V_{\mathrm{DS}}-\frac{1}{2}V_{\mathrm{DS}}^{2})\\ I_{\mathrm{DM}18}=\mu C_{\mathrm{ox}}\frac{W_{18}}{L_{18}}((V_{\mathrm{GSM}18}-V_{\mathrm{TH}})V_{\mathrm{DS}}-\frac{1}{2}V_{\mathrm{DS}}^{2}) \end{array}\right.$$

Four transistors from M11 to M14 form two pairs of current mirrors, which are used to subtract the current of M16 and M18 to obtain compensating current $I_{OUT}$. If transistors M18 and M16 are the same sizes, then the current output of the current compensation of the current source circuit can be expressed as follows:

(10) $$I_{OUT}=M\mu C_{ox}\frac{W_{16}}{L_{16}}\left(V_{GSM16}-V_{VGSM18}\right)V_{DS}$$

where M is the scale factor of the output current of the compensation current source circuit. Substituting Equation (10) into Equation (8) yields the following relationship:

(11) $$V_{HYS}\propto \sqrt{M\frac{W_{16}}{L_{16}}\left(R_{1}-R_{2}\right)R_{3}}$$

By adjusting the coefficient M, different voltage hysteresis values can be achieved (the $V_{HYS}$ does not include μ and $C_{OX}$). With the compensation current source, terms related to the process and temperature, such as $\mu_{P}$ and $C_{OX}$, can be effectively eliminated. In this paper, R1 and R2 are used to provide the gate bias voltage to M16 and M18, respectively. Since M16’s current needs to be slightly greater than that of M18, R1 is set to 180K, and R2 is set to 150 K. R3 provides a bias voltage for the two comparator positive inputs, which is set to 50 K. The constant M in Equation (10) is set to 1.

### 3.3. HCI Monitoring Circuits

**Figure 5:**
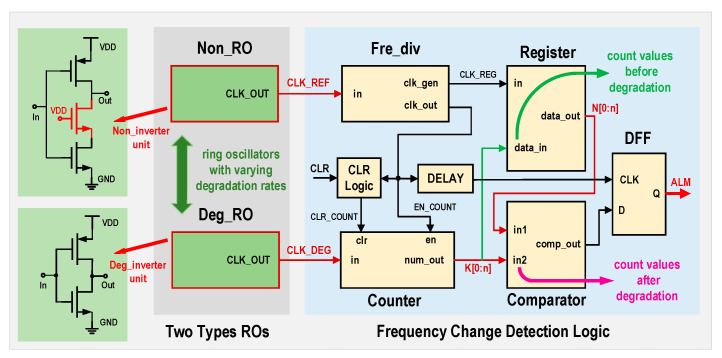
HCI prediction circuit structure diagram.

The HCI prediction circuit structure is shown in Figure 5. The core modules of the circuit are two ring oscillators with different degradation rates. The Non_RO (non-degrading ring oscillator) outputs a frequency that serves as a reference, while the Deg_RO (degrading ring oscillator), which is subject to HCI effects, exhibits a frequency degradation over time, reflecting the aging process of the circuit under prediction. The inverters that make up these two types of ring oscillators differ structurally, with the HCI effect in the Non_RO being partially mitigated. As a result, the output frequency CLK_REF of the Non_RO can be considered invariant over time.

In the initial state, the output frequency of the Deg_RO is counted, and the n+1 bit count value  A[0:n] is stored in the register. Initially, B[0:n]=A[0:n]. As the influence of HCI effect becomes manifest, the count value of the counter for the Deg_RO, denoted by B[0:n], progressively diminishes. Upon reaching a point where B[0:n] has decreased below the initial count value A[0:n] by a predefined magnitude, the digital comparator’s output transitions to a different logic state. This transition is subsequently captured by a flip-flop before the arrival of the subsequent count enable signal, thereby generating an anticipatory alert. The EN_COUNT signal depicted in Figure 5 serves as the enable signal for the counter, derived from the frequency division of CLK_REF. Consequently, it is a periodic square wave signal with a constant frequency that is significantly lower than the frequency of the ring oscillators.

As shown in Figure 5, in this paper, the inverters constituting the non-degrading ring oscillator have an additional normally open NMOS transistor connected between the drain of the bottom NMOS and the output terminal, serving as a voltage divider element. This structure offers two main advantages: 1, Reducing the channel electric field can significantly diminish the impact of HCI in CMOS devices. As a result, the ring oscillator constructed with these inverters will exhibit a more stable frequency. 2, The output frequency of the Non_RO will be lower than that of the Deg_RO. Consequently, in the subsequent frequency change detection logic, the count value of the Deg_RO pulses will be larger, allowing for a more precise detection of frequency variations.

## 4. Results

**Figure 6:**
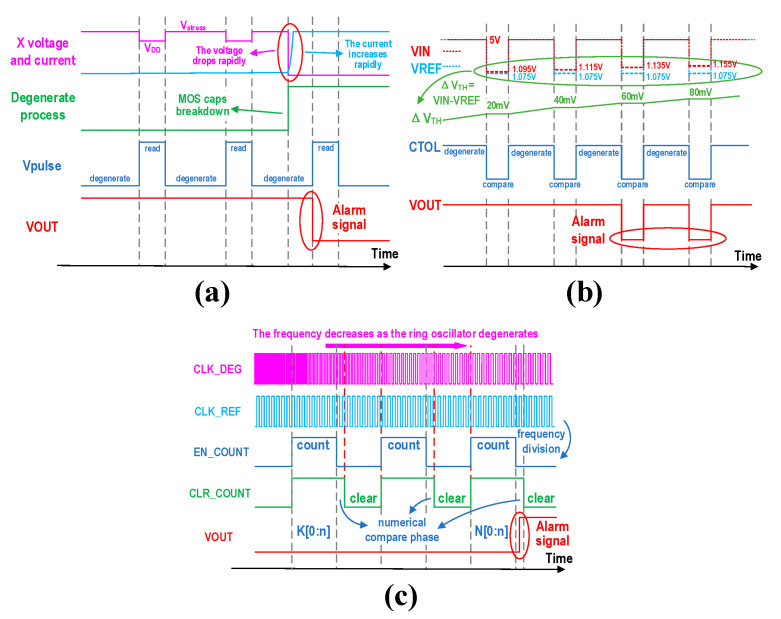
Prediction circuit simulation timing diagram. (**a**) TDDB prediction circuit simulation timing diagram. (**b**) NBTI prediction circuit simulation timing diagram. (**c**) HCI prediction circuit simulation timing diagram.

To intuitively observe the functionality of the detection circuits, we intentionally introduced degraded parameters during simulation to ensure proper functionality in the results. However, such adjustments are not necessary when these circuits are embedded as IPs into the SOC. In Figure 6a, the *Vpulse* signal is an applied signal solely for observing the circuit’s degradation process; similarly, the CTOL signal in Figure 6b serves the same purpose. Figure 6a illustrates the simulation timing diagram of the TDDB prediction circuit. In this diagram, the voltage at node X remains at high when the MOS capacitor has not experienced breakdown. It switches between Vstress and VDD following the high and low levels of Vpulse, with the stress voltage Vstress being slightly higher than VDD. The Vpulse signal is used to control the application of stress and the reading of the MOS capacitor voltage. During the high level of Vpulse, the voltage at node X is read via the latch. The simulation results show that when an MOS capacitor breaks down, the voltage at node X decreases rapidly and the current increases rapidly. In the next read phase, the latch detects the low level at node X and latches the output, which serves as the alarm signal. Figure 6b shows the simulation timing diagram of the NBTI prediction circuit. The power supply voltage is 5V, and CTOL is a continuous square wave with a high duty ratio, being used to control the switch between the degradation state and the detection state. The VIN terminal voltage represents the source and drain voltage of the stressed transistor M1, which is used to reflect the change in the threshold voltage. The VREF terminal voltage represents the source and drain voltage of the reference transistor M4, which does not degrade throughout the process. The difference between VIN and VREF indicates the change in the threshold voltage of M1, reflecting the degree of degradation of M1 under the NBTI effect. According to the simulation results, both VIN and VREF are equal to VDD in the degraded state. When M1 is under stress and M4 is disconnected from the circuit, the difference in threshold voltage ($\Delta V_{TH}$) increases progressively. During the detection phase, both M1 and M4 are connected to the circuit, with the equal current flowing through both branches. As a result, VIN and VREF revert to a lower comparative value, and $\Delta V_{TH}$ remains unchanged. When $\Delta V_{TH}$ exceeds the hysteresis voltage of the comparator, the output VOUT will generate an alarm signal during the subsequent detection phase. Figure 6c shows the simulation timing diagram of the HCI prediction circuit, where CLK_REF and CLK_DEG represent the output clock frequency signals from two distinct types of ring oscillators. Due to the inclusion of anti-degradation NMOS transistors, the output frequency of the non-degrading ring oscillator remains constant. In contrast, the output frequency of the conventional ring oscillator decreases as the circuit ages. The EN_COUNT signal, derived from the frequency division of CLK_REF, serves as the enable signal for the counter and counts the falling edge of CLK_DEG during its high level. The CLR_COUNT signal, generated by CLR_Logic, is the clear signal for the count value. It clears the count value after the comparison of the count values is completed and before the arrival of the next counting enable signal. In the initial state, the count value is set as B[0:n]. As the frequency of CLK_DEG decreases, the count value also decreases. When the count value drops to A[0:n]+Z<B[0:n] (where Z is the predefined frequency degradation threshold), the comparator’s output changes, and an alarm signal is output through the D flip-flop during the count value comparison phase.

**Figure 7:**
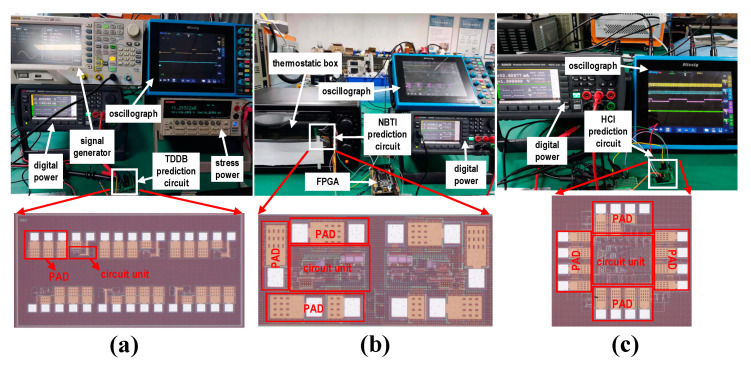
Test environment diagram. (**a**) TDDB prediction circuit test environment. (**b**) NBTI prediction circuit test environment. (**c**) HCI prediction circuit test environment.

**Figure 8:**
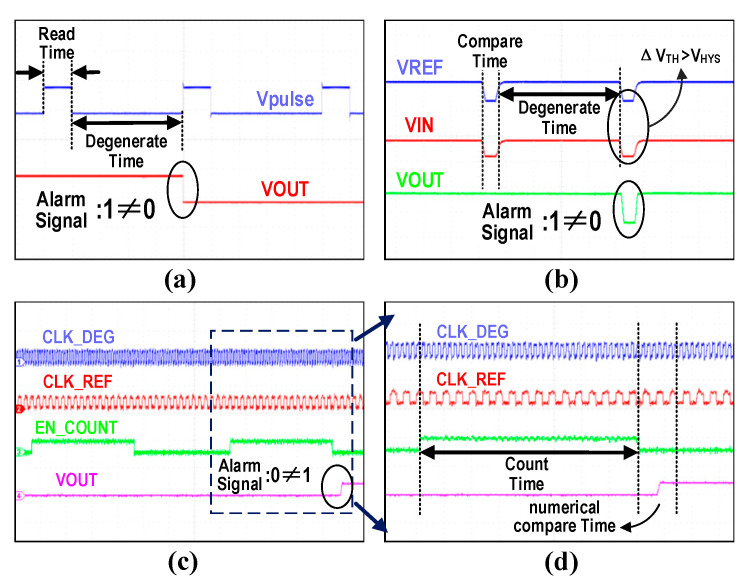
Test results of the prediction circuit. (**a**) TDDB prediction circuit test result. (**b**) NBTI prediction circuit test result. (**c**) HCI prediction circuit test result. (**d**) HCI prediction circuit’s output signals.

The circuit was fabricated using Smartchip’s 0.18 μm CMOS process. The chip testing process primarily focused on conducting accelerated aging tests for the three types of prediction circuits. Figure 7a–c show the test environments for TDDB, NBTI, and HCI. The digital power supply in Figure 7 provides power to the test chip. The temperature-controlled chamber and stress power supply are used to generate accelerated aging stress. The FPGA and signal generator supply the pulse signals required for testing, while the oscilloscope displays the waveforms of key signals within the test chip. The key signal waveform is shown in Figure 8. The waveform diagram in Figure 8a illustrates the transition of the alarm signal for the TDDB prediction circuit. The *Vpulse* signal is generated by the signal generator, and the VOUT signal is the monitoring signal for whether breakdown occurs. During the high level of *Vpulse*, the alarm signal flips from high to low if the MOS capacitor has already degraded, which is consistent with the simulation timing results. The waveform diagram shown in Figure 8b illustrates the alarm signal and the two input signals of the hysteresis comparator for the NBTI prediction circuit. FPGA is used to generate periodic pulse signal CTOL, which is used to detect the difference between VREF and VIN. During the comparison phase, the alarm signal transitions from high to low if the difference in threshold voltages increases to a certain extent ($\Delta V_{TH}>V_{HYS}$), which is consistent with the simulation timing results. Figure 8c displays the waveform diagram for the outputs of the two ring oscillators, the counter enable signal, and the output signal in the HCI prediction circuit. The whole HCI monitoring circuit does not require any external signal inputs. Figure 8d is a zoomed-in section of Figure 8c, highlighting the details of the HCI prediction circuit’s output signals. During the comparison phase, when the count value decreases to the degradation threshold, the alarm signal transitions from low to high, consistent with the simulation timing results.

The work of this paper is compared with other studies, as shown in Table 1.

## 5. Conclusions

This paper presents the designs of three types of reliability failure prediction unit circuits that can be embedded in SoCs, reducing the steps required by traditional life prediction methods that rely on accelerated aging tests. The test results indicate that in the TDDB monitoring circuit, the static power consumption is only due to the capacitor charge leakage current, which is negligible in large SoC chips. In the NBTI monitoring circuit, power consumption depends on the clock frequency of the CTOL signal, and the measured results show an average power consumption of 26.3 μA@60 kHz. For the HCI monitoring circuit, power consumption depends on the ring oscillator frequency. In this paper, the method is only used for functional verification, and the ring oscillator frequency is at the 12 MHz level, with the overall monitoring circuit power consumption being 1.25 mA. If applied to a SoC system, the frequency should be adjusted to reduce power consumption. All three monitoring circuits can be embedded as IP cores into any SoC module to monitor specific transistors or circuits prone to aging. In the Smartchip CMOS 0.18 μm process, the total area consumed by the monitoring module is 57 μm × 41 μm + 320 μm × 132 μm + 323.845 μm × 366.39 μm. This study designed three monitoring circuits suitable for use as IPs, and individual testing results demonstrated that all three circuits functioned correctly. When applied to SoC failure monitoring, these circuits can be independently embedded based on the modules in the SoC that are most prone to failure.

## Figures and Tables

**Table 1:** Summary of work and comparison with other papers.

Reference	[11]	[12]	[13]	[14]	[16]	This Work
Process (nm)	Off chip	65	45	90	-	180
Operating voltage (V)	1.0–1.5	1.2	1	1	1.2	1.8/5
Temperature range	−30~115 °C	27 °C	25~125 °C	-	27~127 °C	−40~120 °C
Monitoring type	HCI and NBTI	None	NBTI	HCI and BTI	HCI and NBTI	All
Embedded in SOC	No	Yes	Yes	Yes	Yes	Yes
Real-time monitoring	No	Yes	Yes	Yes	Yes	Yes
Consider process variations	No	Yes	No	No	Yes	Yes

## Data Availability

The original contributions presented in the study are included in the article, further inquiries can be directed to the corresponding author.
